# FRMDB: Face Recognition Using Multiple Points of View

**DOI:** 10.3390/s23041939

**Published:** 2023-02-09

**Authors:** Paolo Contardo, Paolo Sernani, Selene Tomassini, Nicola Falcionelli, Milena Martarelli, Paolo Castellini, Aldo Franco Dragoni

**Affiliations:** 1 Dipartimento di Ingegneria dell’Informazione, Università Politecnica delle Marche, Via Brecce Bianche 12, 60131 Ancona, Italy; 2 Gabinetto Interregionale di Polizia Scientifica per le Marche e l’Abruzzo, Via Gervasoni 19, 60129 Ancona, Italy; 3 Department of Law, University of Macerata, Piaggia dell’Università 2, 62100 Macerata, Italy; 4 Dipartimento di Ingegneria Industriale e Scienze Matematiche, Università Politecnica delle Marche, Via Brecce Bianche 12, 60131 Ancona, Italy

**Keywords:** face verification, face identification, video surveillance, police mugshots, law enforcement

## Abstract

Although face recognition technology is currently integrated into industrial applications, it has open challenges, such as verification and identification from arbitrary poses. Specifically, there is a lack of research about face recognition in surveillance videos using, as reference images, mugshots taken from multiple Points of View (POVs) in addition to the frontal picture and the right profile traditionally collected by national police forces. To start filling this gap and tackling the scarcity of databases devoted to the study of this problem, we present the Face Recognition from Mugshots Database (FRMDB). It includes 28 mugshots and 5 surveillance videos taken from different angles for 39 distinct subjects. The FRMDB is intended to analyze the impact of using mugshots taken from multiple points of view on face recognition on the frames of the surveillance videos. To validate the FRMDB and provide a first benchmark on it, we ran accuracy tests using two CNNs, namely VGG16 and ResNet50, pre-trained on the VGGFace and VGGFace2 datasets for the extraction of face image features. We compared the results to those obtained from a dataset from the related literature, the Surveillance Cameras Face Database (SCFace). In addition to showing the features of the proposed database, the results highlight that the subset of mugshots composed of the frontal picture and the right profile scores the lowest accuracy result among those tested. Therefore, additional research is suggested to understand the ideal number of mugshots for face recognition on frames from surveillance videos.

## 1. Introduction

Artificial Intelligence (AI) and Deep Learning (DL) have allowed major advancements in different application domains [1]. The law enforcement sector is one such domain, leveraging AI and DL to serve crime investigations [2] by implementing applications increasingly able to autonomously detect suspicious activities [3]. For example, in applications such as violence detection [4,5], weapon detection [6,7], traffic accident detection [8], and human trajectory prediction [9], DL-based techniques exploit the availability of video surveillance systems, providing accurate and rich information to achieve security [10].

As one of the most natural biometric techniques for identification [11], face recognition can be considered a law enforcement application. In fact, the natural variation among individuals leads to good inter-class separation making the facial characteristics appealing for biometric recognition [12]. Whereas early face recognition methodologies were based on Principal Component Analysis (PCA) and Linear Discriminant Analysis (LDA) (i.e., Eigenfaces [13] and Fisherfaces [14]), face recognition became mature with the results achieved by Convolutional Neural Networks (CNNs). CNNs were successfully applied in face verification, i.e., the job of assessing whether two face images belong to the same person, and identification, i.e., the job of assessing whether a face image belongs to a specific identity in a set of known subjects [15]. Thanks to such advancements, face recognition is currently used for biometric authentication in applications such as smartphone unlocking [16] and passport verification [17].

Furthermore, face recognition is considered mature enough to integrate Automated Fingerprint Identification Systems (AFISs) when only the images of a person suspected of a crime are available, instead of fingerprints [18]. As an example, the SARI (“Sistema Automatico Riconoscimento Immagini”) system implemented by the Italian Police supports the work of an operator in finding a possible correspondence between a face image of an unknown subject and a known identity belonging to the AFIS mugshots database [19]. The correspondence is an investigative clue despite cannot be used as forensic evidence in court.

The integration of face recognition in existing decision support systems for crime investigation, such as the SARI, demonstrating its readiness level. Nevertheless, despite the progress in Pose-Invariant Face Recognition (PIFR), i.e., the identification or verification of individuals with face images captured under arbitrary poses, matching between two arbitrary poses still is an open challenge [20,21]. Moreover, in scientific literature, there is a lack of research on face recognition systems that can be used for identification or verification by comparing images taken from CCTV with the available database of mugshots [22]. Furthermore, during the photo-signaling procedure, national police forces routinely collect two pictures, i.e., the frontal picture and the right profile (commonly known as mugshots), together with the fingerprints and personal information of a subject. However, there is a lack of research directed at understanding to what extent CNNs for face recognition is effective in identifying a known person in video surveillance clips when only the two standard images of photo-signaling are available as reference pictures [23].

To this end, this paper extends our previous work [24] by proposing the Face Recognition from Mugshot Database (FRMDB), a dataset of face images and videos to test the use of mugshot pictures taken from multiple Points of View (POVs), as reference images in the face recognition on video surveillance frames. The proposed dataset can be used to measure the accuracy of face recognition with different subsets of mugshot pictures. The goal is to understand if using face images from multiple POVs can positively impact face recognition performance, justifying the effort needed to take more pictures and store them. Specifically, this paper adds the following original contributions to the state-of-the-art of face recognition:

- It proposes a novel dataset, the FRMDB, composed of 39 subjects with mugshots taken from 28 different perspectives plus 5 surveillance videos taken from 5 different perspectives. The dataset is open-access and freely released in a GitHub repository (the proposed dataset is available at: https://github.com/airtlab/face-recognition-from-mugshots-database, accessed on the 30 December 2022).
- It presents a literature review of existing databases for face recognition, analyzing their potential in benchmarking techniques for verification and identification in surveillance scenarios. Although existing surveys and reviews about face recognition also include a detailed description of available databases, such as in [25,26,27,28], we analyze datasets considering the availability of images and clips suitable to test recognition in video surveillance conditions.
- It compares the results of two well-established CNNs for face recognition on the proposed dataset and the Surveillance Cameras Face (SCFace) database [29]. Such comparison is useful to validate the goal of the FRMDB, i.e., testing face recognition on security camera frames when different mugshots are available for the identification.
- It provides an initial benchmark for the proposed dataset, starting to analyze the performance of the face recognition when different subsets of mugshots, taken from various POVs, are available as a reference. The source code of the experiments is published in an open-access GitHub repository (the source code of the tests is available at: https://github.com/airtlab/tests-on-the-FRMDB, accessed on the 30 December 2022).

In fact, as analyzed in Section 2, despite the availability of many databases for face verification and identification, the SCFace is the only one including mugshots and surveillance camera images that can perform face recognition in CCTV frames using pictures from nine different points of view as reference images. However, all the faces in the surveillance camera frames are almost frontal. Therefore, we built a new dataset, the FRMDB, containing more mugshots for each subject (28) and videos from surveillance cameras taken from five different points of view. The FRMDB is specifically tailored to presenting a set of mugshots systematically taken from multiple points of view. The videos from the security cameras currently contain the same lighting and do not include occlusions. Instead, the background clutter is different for each of the five points of view, as described in Section 3.

Concerning the tested CNNs, we compared VGG16 [30] and ResNet50 [31], pre-trained on the VGGFace [32] and VGGFace2 [33] datasets for the extraction of facial features.

In addition, by testing these CNNs on the SCFace database and the proposed dataset, we aim at understanding the impact that different sets of mugshots might have on the identification of suspected subjects recorded in the security camera footage. The mugshots are taken from multiple points of view, beyond the standard frontal and profile pictures collected by police forces during the photo-signaling procedure. Moreover, the results reported in this paper are fully reproducible, given that both the proposed dataset and the source code of the tests are published in dedicated open-access GitHub repositories.

The rest of the paper is organized as follows. Section 2 includes a literature review of the datasets available for face recognition, highlighting the differences from the one proposed in this paper, and face recognition techniques, justifying the choice of CNNs for our comparison. Section 3 describes the dataset built for our research and the methodology implemented to run our comparative tests. Section 4 presents the results of our tests, analyzing the accuracy performance of the SCFace database and our dataset using varying sets of mugshots as reference pictures. Finally, Section 5 provides the conclusions of this research and suggests future works.

## 2. Literature Review

To explain the need for a new dataset and justify the choice of the CNNs used in the experiments, we describe the features of the face databases available in the literature (Section 2.1) and present the evolution of face recognition techniques over the years (Section 2.2). Although several databases are available, including some for masked face recognition that has recently appeared [34,35], most do not include features adequate to evaluate the recognition performance in clips from security cameras, using as reference images sets of mugshots different from the frontal and profile pictures taken during the standard photo-signaling procedure. Nevertheless, CNN-based techniques demonstrated their superiority, where conditions such as lighting, facial expression, and pose are not fixed [15,28]. For these reasons, we propose a new dataset and compare two different CNNs on it.

### 2.1. Databases for Face Recognition

Given that face recognition has attracted the interest of computer vision researchers for over forty years, several databases of face images are available to benchmark the different techniques. One of the first databases appeared to compare different recognition methodologies is the AT&T Database of Faces, formerly known as the ORL (Olivetti Research Laboratory) Database of Faces [36]. Despite including 10 different grayscale images (92 × 112 pixels) for each of the 40 subjects included in the database, varying the facial expressions and the lighting, all the images are in frontal position, without security videos or frames from security cameras to compare with. The database was free to use and open-access, even if, at the time of writing, the official website seems discontinued.

As the face recognition techniques improved and obtained outstanding results on the AT&T database and similar datasets, the research focused on unconstrained scenarios, i.e., with varying conditions concerning ambient illumination, image resolution, background clutter, facial pose, expression, and occlusion [37]. Therefore, databases of face images dedicated to unconstrained face recognition appeared, such as the Labeled Face in the Wild (LFW) [38,39] and the YouTube Faces Database [40]. The LFW database includes 13,233 color images (250 × 250 pixels) of 5749 unique people, with 1680 subjects having two or more images. The face images were collected from various sources on the web using the Viola–Jones face detector [41]. The LFW database is free to use and open-access. However, the LFW database is intended for unconstrained face verification, and therefore, it does not include systematically taken sets of mugshots and videos to compare with. As such, it is not adequate to evaluate the performance of face recognition techniques by testing pictures from multiple points of view. Likewise, the YouTube Face Database includes 3425 color YouTube videos of 1595 different people. Thus, even such a database is for unconstrained face verification without sets of systematically taken mugshots to be used in video surveillance scenarios. Similarly to the LFW, the YouTube Faces Database is free to use and open-access.

With the results achieved by CNNs in image recognition and face recognition, databases with more face images and unique identities appeared, to the point that training and evaluation of CNNs on the scale of the millions is possible. To this end, the CASIA-Webface database [42] includes 494,414 face images of 10,575 unique identities. The images are crawled from the web at various resolutions. The database is available upon request, even if the official website seems to be discontinued at the time of writing. The Megaface Challange Dataset [43,44] includes 4.7 million color photos of 672,057 unique subjects at various resolutions. As the Megaface Challenge ended, the database was discontinued and Megaface data were no longer officially distributed. The VGGFace Dataset [32] contains 982,803 color images (95% frontal, 5% profile) of 2622 unique identities, whereas the VGGFace2 Dataset [33] includes 3.31 million color images of 9131 unique subjects. Both the VGGFace and the VGGFace2 datasets are free to use and open-access. The Megaface Challenge, the VGGFace, and the VGGFace2 datasets include faces collected from the web under different conditions of lighting, pose, expression, and occlusion, similar to the LFW and YouTube Face Datasets. The amount of images available in such databases make them ideal for training DL-based techniques such as the CNNs, even to be used in a transfer learning fashion, as we did with the VGGFace and VGGFace2 datasets in this paper. However, given that these databases do not include systematically taken sets of mugshots and security videos to compare with, they are not suitable for evaluating the impact of the use of mugshots from multiple POVs in the face recognition performance in surveillance scenarios.

Over the years, some databases, including subjects with different poses, i.e., mugshots from multiple perspectives, have also been published. For example, the Facial Recognition Technology (FERET) database [45,46] includes 14,051 color images (512 × 768 pixels) of 1199 subjects. For 200 subjects among those composing the database, 9 mugshots systematically taken from different points of view are available (from −60${}^{\circ }$ to +60${}^{\circ }$). The dataset is available upon request with a dedicated release agreement. Similarly, the Max Planck Institute for Biological Cybernetics Face Database (MPI Database) [47] includes color images (256 × 256 pixels) taken from 7 different points of view (from −90${}^{\circ }$ to 90${}^{\circ }$, with a 30${}^{\circ }$ step) about 200 unique identities, for a total of 1400 images. However, the database is no longer available. The Extended Yale Face Database B [48,49] includes 16,128 grayscale images (640 × 480 pixels) of 28 unique identities obtained by combining 9 different poses (a frontal face, 5 pictures at 12${}^{\circ }$, and 3 pictures at 24${}^{\circ }$) with 64 different lighting conditions. The database is free to use and open-access. The Korean Face Database (KFDB) [50] also includes face images from different points of view. Specifically, it has 52,000 color images (640 × 480 pixels) of 1000 unique subjects, with varying lighting conditions and facial expressions and is systematically taken from 7 different angles (from −45${}^{\circ }$ to +45${}^{\circ }$, with a 15${}^{\circ }$ step). At the time of writing, the database is not available. The CAS-PEAL database [51] contains 30,900 color images (360 × 480 pixels) of 1040 unique identities. Facial images from 21 different points of view are available, combining 7 different angles on the horizontal plane (from −67.5${}^{\circ }$ to +67.5${}^{\circ }$, with a step of 22.5${}^{\circ }$) and 3 different angles on the vertical plane (from −30${}^{\circ }$ to +30${}^{\circ }$, with a step of 30${}^{\circ }$). For some subsets of the subjects, other images with different facial expressions and lighting and wearing varying accessories are available. The database is available upon request. The Multi-PIE dataset [52] contains 755,370 color images (3072 × 2048 pixels) of 337 unique subjects recorded in different sessions to include pose, illumination, and expression variations. For each session, 13 images ranging from −90${}^{\circ }$ to +90${}^{\circ }$ with a step of 15${}^{\circ }$ on the horizontal plane were taken by different cameras placed at head height. Two additional images at ±30${}^{\circ }$ on the horizontal plane and above the head height were taken. The dataset is available for distribution upon request. The NIST Mugshot Identification Database (MID) [53] includes 3228 grayscale images (of varying sizes) of 1573 individuals. A total of 1333 subjects have both the front and profile mugshots, 131 subjects have two or more frontal pictures and 89 subjects have two or more profile pictures. The database is available upon request. Despite the fact that the FERET, Yale, MPI, KFDB, CAS-PEAL, Multi-PIE, and MID databases include mugshots from multiple points of view, they do not contain any frame or video of the subjects from security cameras to allow analyzing the impact of using different subsets of pictures from different angles in the face recognition performance in surveillance scenarios.

The ChokePoint Dataset [54] differs from the aforementioned datasets. With two sets of subjects of 25 and 29 (the sets overlap), and 48 video sequences, the dataset is intended to reproduce video surveillance conditions for video-to-video verification. However, the dataset does not include mugshots to be used in the identification or verification of subjects in the videos. The dataset is open-access.

To the best of our knowledge, the only database including mugshots which are systematically taken from multiple points of view and face images from security cameras is the Surveillance Cameras Face (SCFace) database [29]. In fact, the database contains 4160 images of 130 unique subjects. Each subject has 9 color mugshots (2048 × 3072 pixels) taken from −90${}^{\circ }$ to +90${}^{\circ }$ with a step of 22.5${}^{\circ }$, another color frontal mugshot (2048 × 3072 pixels), an InfraRed (IR) frontal mugshot (320 × 426 pixels), and 21 images (15 color images and 6 IR images) of varying size taken with seven security cameras at three different distances. The database is available upon request, with a dedicated release agreement. Given its features, the SCFace database was the only one available to test the capability of CNNs to perform face recognition on surveillance images using different subsets of mugshots, as we did in our previous research [24]. Nevertheless, all the pictures from the security cameras are almost frontal, whereas, in real life, a subject might be framed from different perspectives.

Whereas some of the existing datasets, such as VGGFace and VGGFace2, allow training DL-based techniques to extract face features, most of the face databases are not adequate to assess the recognition capabilities on video surveillance clips when different sets of mugshots, from multiple points of view, are available as reference images. To tackle such limitation, we propose a new dataset that includes 39 subjects, with 28 mugshots pictures and 5 videos taken from security cameras placed in five different spots. The mugshot pictures are taken by combining 7 angles on the horizontal plane (from −135${}^{\circ }$ to +135${}^{\circ }$ with a step of 45${}^{\circ }$) and 4 angles on the vertical plane (from −60${}^{\circ }$ to +30${}^{\circ }$ with a step of 30${}^{\circ }$). Therefore, as shown in Table 1, which summarizes the features of the databases discussed in this subsection, the proposed dataset allows the use of mugshots from multiple POVs on both the horizontal and vertical plane for the identification of subjects in clips from security cameras. We report the details of the proposed dataset in Section 3.1.

### 2.2. Evolution of Face Recognition Techniques

The first techniques for the automatic recognition of faces in digital images were based on Principal Component Analysis (PCA) and Linear Discriminant Analysis (LDA). Specifically, Turk and Pentland [13] proposed computing the Eigenfaces, i.e., to extract a vector of features that maximize the inter-class variance in a set of training images. By projecting a face image in the space obtained with the PCA, face identification can be performed with the nearest neighbor method, computing the distance from training images. Instead, Belhumer et al. [14] proposed adding Linear Discriminant Analysis (LDA) to the PCA in order to minimize intra-class variance, calling this technique Fisherfaces. Differently from Eigengaces and Fisherfaces, Ahonen et al. [55] compute Local Binary Patterns Histograms (LBPH) on face images to describe face regions with Local Binary Patterns (LBP). In this way, a distance function based on LBPHs can be used to perform face identification. Despite exhibiting promising results in databases such as the AT&T, where some variables among pose, expression, and lighting are fixed, these techniques are insufficient to extract features invariant to real-world changes [56], such as in video surveillance clips.

After the outstanding results in image processing from the performance by AlexNet in the 2012 ImageNet competition [57], CNNs also exhibited robust results in face recognition with the changing conditions of lighting, expression, and pose typical of unconstrained face images [15]. For example, DeepFace [58], an eight-layer CNN used to process three channels of 152 × 152 face images, obtained a 97.35% accuracy on the LFW dataset [59]. Parkhi et al. [32] trained VGG16 [30], a 16-layer CNN, on the VGGFace dataset and tested it on the LFW dataset, obtaining a 98.95% accuracy. Similarly, FaceNet [60], a 22-layer CNN trained in several experiments with a varying number of face images, between 100 and 200 million, belonging to 8 million different subjects, obtained a 99.63% accuracy on LFW, using 220 × 220 input images. Cao et al. [33] tested ResNet50 [31], a 50-layer CNN based on residual learning, and SE-ResNet50 (i.e., ResNet50 with the Squeeze and Excitation blocks [61]) on the VGGFace2 dataset, obtaining a top-one identification error of 3.9% with ResNet50 on the VGGFace2 dataset. You et al. [62] compared different CNNs on the LFW dataset (and on other datasets) applying transfer learning: the CNNs were pretrained on the CASIA-Webface database. The best models were VGG16 [30] and ResNet50, who obtained 98.94% and 98.52% accuracy on the LFW database. Furthermore, although Pose-Invariant Face Recognition (PIFR), i.e., the identification or verification of individuals with face images captured under arbitrary poses, is still an open challenge, recent techniques have shown encouraging progress [20,21]. Most of these techniques are based on the generation of synthetic (or partially synthetic) images to frontalize the face or create pictures at any pose. For example, Hassner et al. [63] proposed aligning the facial feature points, despite the subject pose, to a 3D face surface unique for all the faces. By back-projecting the color of the face picture to the 3D surface and borrowing appearances from corresponding symmetric sides of the face, they produce the final frontal image. They tested their methodology on the LFW dataset, obtaining a 91.62% accuracy. Tran et al. [64,65] presented an extension of Generative Adversarial Networks (GAN) to generate an arbitrary number of synthetic faces at any pose. They obtained 90.8% identification accuracy on the Multi-PIE dataset. Zhao et al. [66] introduced a Pose Invariant Model (PIM) based on the use of a GAN for the face frontalization and CNN for the learning of face features. Testing the recognition accuracy on the images of the Multi-PIE dataset at ±15${}^{\circ }$, ±30${}^{\circ }$, ±45${}^{\circ }$, ±60${}^{\circ }$, ±75${}^{\circ }$, and ±90${}^{\circ }$, they obtained an average of 96%. These methodologies have been applied to datasets for unconstrained face recognition, without considering comparing mugshot pictures with frames from surveillance cameras. Instead, in the proposed dataset, we combined these two aspects: this can be useful to benchmark traditional CNNs (without frontalization and synthetic images) as in our approach or the PIFR techniques listed.

Given the capabilities demonstrated by CNNs on face recognition, in this paper, we compare VGG16 and ResNet50 as the first step in the evaluation of the recognition capabilities when using different subsets of mugshots as reference images for the task.

## 3. Materials and Methods

Given the need for face databases to assess the capability of recognizing faces in security frames from mugshots taken from multiple points of view, we propose a new dataset including 28 different mugshots plus 5 videos from security cameras for each subject. Such a dataset will be helpful to evaluate if using more poses, in addition to the frontal picture and the right profile usually available in the databases of law enforcement agencies, as it can have a positive impact on face recognition performance. To set an initial benchmark for the proposed dataset, we tested two different CNNs, namely VGG16 and ResNet50, pre-trained for face recognition on large face databases, i.e., VGGFace and VGGFace2. To this end, Section 3.1 describes the proposed dataset of face images, Section 3.2 gives the details of the CNNs used for our tests, and Section 3.3 explains the experimental protocol and the metrics computed in our tests.

### 3.1. The Proposed Dataset

The Face Recognition from Mugshots Database (FRMDB) includes 39 unique identities, 17 females and 22 males. The average age of the subjects is 24.6, with the youngest individual being 19 years old and the oldest 52 years old (standard deviation 7.8). For each subject, the dataset includes:

- A total of 28 mugshots, i.e., 28 color pictures taken from different points of view with the subject posing during the acquisition.
- A total of 5 security cameras videos, taken from 5 points of view. In addition, a mosaic video including all 5 clips at the same time is available.

**Figure 1:**
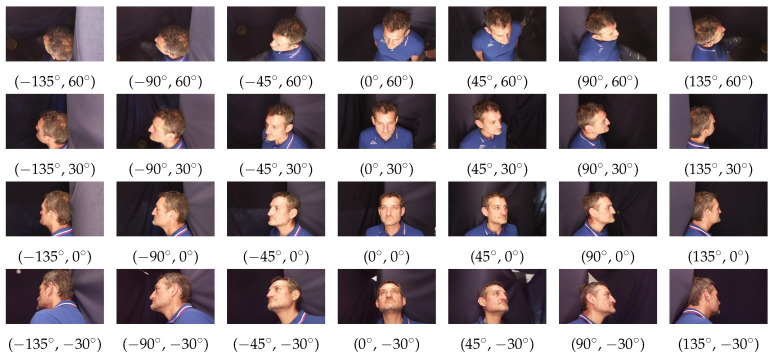
A sample of the mugshots available for each subject in the FRMDB. For each mugshot, the angles from which the picture was taken are reported as a couple (h,v): *h* is the angle on the horizontal plane from −135${}^{\circ }$ to +135${}^{\circ }$, with an increment of 45${}^{\circ }$ between an angle and its adjacent (from left to right); *v* is the angle on the vertical plane from 60${}^{\circ }$ to −30${}^{\circ }$, with a step of −30${}^{\circ }$ between an angle and its adjacent (from top to bottom).

**Figure 2:**
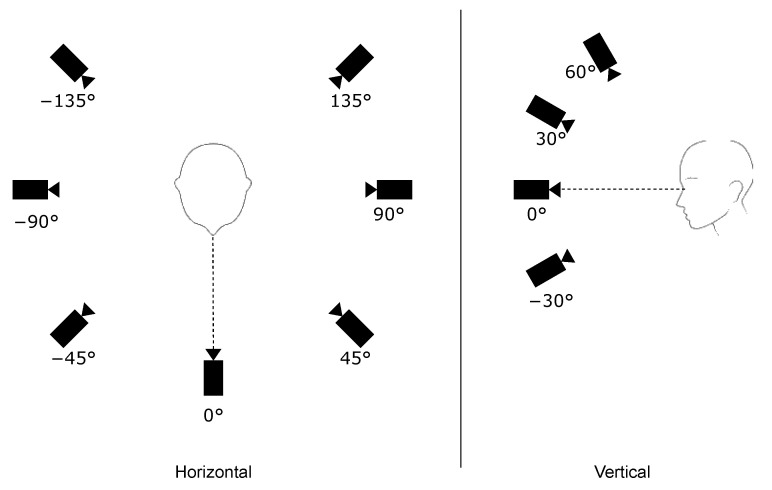
The different points of view for the acquisition of the FRMDB mugshots on the horizontal plane (**left**) and the vertical plane (**right**).

Figure 1 includes the 28 mugshots of the subject “031” in the database (each identity is a 3-figure code to preserve anonymity). Each mugshot is a 972 × 544 pixels JPEG image. We collected the mugshots by taking pictures from 7 angles on the horizontal plane and 4 angles on the vertical plane. Specifically, on the horizontal plane, the pictures were taken from −135${}^{\circ }$ to +135${}^{\circ }$, with a step of 45${}^{\circ }$ (with 0${}^{\circ }$ being in front of the subject). On the vertical plane, the pictures were taken from −30${}^{\circ }$ to +60${}^{\circ }$ (with 0${}^{\circ }$ being the camera on the plane of the subject’s eyes) with a 30${}^{\circ }$ step. In this regard, Figure 2 shows the different points of view on the horizontal and vertical planes used to take the mugshots.

For the experiments presented in this paper, we manually cropped the face in each mugshot for each subject. Therefore, we published the cropped mugshots in the dataset repository.

**Figure 3:**
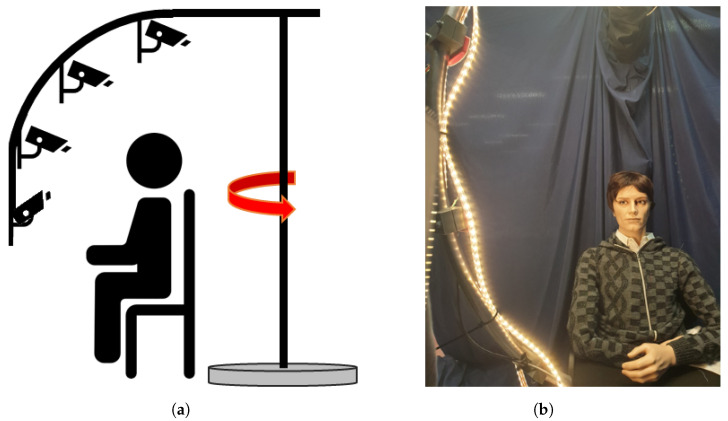
The robotic arm used to collect the mugshots for each subject in the FRMDB. The cameras placed on a rotating arm (**a**) took four pictures with 4 different angles on the vertical plane (from −30${}^{\circ }$ to +60${}^{\circ }$) at 7 different positions (from −135${}^{\circ }$ to +135${}^{\circ }$) on the horizontal plane; the rotating axis is evidenced by the red arrow. The subject was sitting in the middle, and the lighting was provided by a led strip placed on the rotating arm (**b**).

To take the mugshots, we asked the subject to sit against a dark background, and the pictures were taken by 4 cameras placed in 4 spots on a robotic arm, which was rotating around the vertical axis, as showed in Figure 3. Such rotation allowed the acquisition of the mugshots from the 7 angles on the horizontal plane and 4 angles on the vertical plane. In this way, the pictures were taken four by four: all the pictures with the same horizontal angle, i.e., in the same rotation position (in the same column in Figure 1), were taken at the same time. The lighting was provided by a led strip placed on the rotating arm.

**Figure 4:**
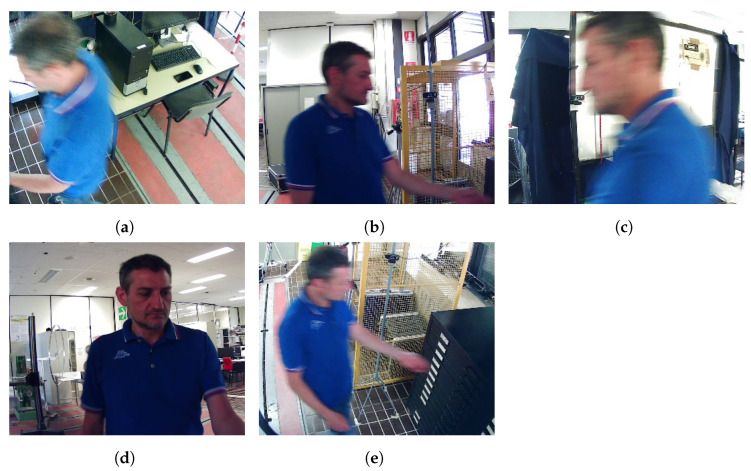
Frames from the videos of the security cameras in the proposed database. The videos were recorded at the same time from 5 different points of view (**a**–**e**). During the recording of the videos, the subjects were asked to walk to a chest of drawers, open a drawer, extract a paper, sign the paper, and go back to the starting spot.

Figure 4 includes a frame for each of the 5 security camera videos belonging to subject “031” in the database. The videos are encoded with the H.264 codec (the container format is Matroska—mkv) and recorded at 60 frames per second. The frame size is 352 × 288 pixels (the size of the mosaic, including all the 5 clips is 1280 × 720 pixels). The average duration of the videos is 18.5 s (minimum 15 s, maximum 29 s, standard deviation 2.9 s). To record the security camera videos, each subject was asked to walk to a chest of drawers, open a drawer, extract a paper, sign the paper, and go back to the starting spot. During the fulfillment of such tasks, 5 cameras placed in 5 different spots recorded the subject. The 5 videos of each subject were recorded at the same time. Although all the security videos were filmed in the same room, with constant lighting, the background clutter depends on the point of view of each camera, as shown in Figure 4.

For the experiments presented in this paper, we manually selected one frame for each video and cropped the face to test the recognition performance on such frames using different sets of mugshots. The selected frames and the cropped faces are available in the proposed dataset repository.

In addition to the described mugshots and videos of the security cameras, the FRMDB includes:

- An additional frontal picture (1920 × 1080 pixels, JPEG) for each subject, taken with a different light from a camera placed in front of the subject.
- For 12 out of the 39 subjects, a second set of 5 videos from the security cameras (plus the mosaic) is available. For these subjects, the second set of the security videos varies because the subject wears different accessories on their head, such as glasses, sunglasses, hats, and bandanas. The subjects do not wear such accessories in the mugshots.
- For 3 out of the 39 subjects, a second set of 28 mugshots taken with the subject smiling.
- A text file for each subject containing the subject’s sex, age, and the accessories worn in the second set of security videos, if available.

These files might be useful for additional recognition tests under varying conditions. However, we did not use such files in the experiments presented in this paper.

### 3.2. The Compared CNNs

Using the proposed dataset and the SCFace database, we tested the recognition capabilities of two different CNNs, namely VGG16 and ResNet50, when different subsets of mugshots were used as the reference images. Specifically, the CNNs extract a face embedding for each face, i.e., a vector of features describing the face image. The embeddings of the mugshots and those of the faces in the security cameras can be compared by means of a distance or similarity measure, such as the Euclidean distance or cosine similarity for face identification and verification.

Concerning VGG16, we used the same architecture as [32], whereas for ResNet50, we used the architecture described in [33]. Specifically, in both networks, the input is a 224 × 224 face image, and the embedding is computed by applying the Global Average Pooling on the output of the last convolutional block of the network. This means that with VGG16, the embedding is a 512-element feature vector, whereas for ResNet50, it is a 2048-element feature vector. Following the results obtained by [33], we L2-normalized the embeddings computed with both CNNs.

The training of the networks is the same as described in [32] for the VGG16 model and in [33] for the ResNet50 models. Therefore, VGG16 was trained from scratch on the VGGFace dataset, using the triplet loss function and the Stochastic Gradient Descent (SGD) for optimization, with batches of 64 samples and the starting learning rate equal to 0.01, decreased three times by a factor of 10 when the accuracy on the validation set stopped increasing. ResNet50 was trained from scratch on the VGGFace2 dataset, using the soft-max loss function and the SGD for optimization, with batches of 256 samples and the starting learning rate equal to 0.1, decreased two times by a factor of 10 when the error stopped decreasing. Instead of re-running the training, we applied the original network weights (the original VGG16 weights can be found at https://www.robots.ox.ac.uk/~vgg/software/vgg_face/, accessed on the 29 December 2022), (the original ResNet50 weights can be found at https://github.com/ox-vgg/vgg_face2, accessed on the 29 December 2022) using the Keras conversion of the original Caffe models (the Keras conversion of the CNNs is available at https://github.com/rcmalli/keras-vggface, accessed on the 29 December 2022).

### 3.3. Experimental Protocol and Evaluation Metrics

We tested the recognition capability of VGG16 and ResNet50, trained on VGGFace and VGGFace2, on the images of the SCFace database and the proposed dataset. Specifically, we defined different subsets of mugshots to be used as reference images for the recognition of faces in the security camera pictures, executing tests on both datasets. In this regard, the complete description of the SCFace database can be found in [29]: the database contains 9 (posed) mugshots of 130 unique subjects; furthermore, the database contains extra 21 face images for each unique subject. Such extra images were cropped from the frames of security camera clips. As explained in Section 2, we consider the SCFace database the most suitable (in addition to the proposed dataset) to understand how the use of mugshots taken from multiple POVs can impact the face recognition performance. The reason is that the 9 mugshots available for each unique subject are systematically acquired from different angles on the horizontal plane, from an angle equal to −90${}^{\circ }$ to 90${}^{\circ }$ (i.e., from the left profile to the right profile, with 0${}^{\circ }$ being the frontal picture) with steps of 22.5${}^{\circ }$ between an angle and the next one.

Table 2 lists the different subsets of mugshots used as images for the comparison to recognize the pictures in the security cameras of the FRMDB and the SCFace database. For each database, the table describes the angles from which the mugshots were taken as a couple (h,v), where *h* is the angle on the horizontal plane and *v* is the angle on the vertical plane. For the SCFace database, the *v* angle is always 0${}^{\circ }$, as different angles on the vertical plane are not available.

In the following, we describe the subsets of mugshots used for face recognition:

- The “Test F” subset, composed of the frontal picture only, i.e., the one at (0${}^{\circ }$, 0${}^{\circ }$) for both databases. The name “Test F” comes from the SCFace database, where “F” is the label given to the frontal pictures.
- The “Test F-L1-R1”, composed of the frontal picture, the left angle nearest to the frontal picture (which is (−22.5${}^{\circ }$, 0${}^{\circ }$) for the SCFace database and (−45${}^{\circ }$, 0${}^{\circ }$) for the FRMDB), and the right angle nearest to the frontal picture (SCFace: (22.5${}^{\circ }$, 0${}^{\circ }$); FRMDB: (45${}^{\circ }$, 0${}^{\circ }$)). The name “Test F-L1-R1” comes from the SCFace database, as F, L1 and R1 are the image labels used for the included pictures.
- The “Test 1” subset, composed of the frontal picture and the right profile picture, i.e., (90${}^{\circ }$, 0${}^{\circ }$), for both databases. This subset reproduces the only mugshots currently available in the database of most police forces.
- The “Test 2” database, composed of the frontal picture, the right profile picture, and the left profile, i.e., (−90${}^{\circ }$, 0${}^{\circ }$).
- The “Test 2” pictures plus the pictures one step closer to the frontal picture starting from the right profile and left profile, which are (77.5${}^{\circ }$, 0${}^{\circ }$) and (−77.5${}^{\circ }$, 0${}^{\circ }$) for the SCFace database, and (45${}^{\circ }$, 0${}^{\circ }$) and (−45${}^{\circ }$, 0${}^{\circ }$) for the FRMDB. We called these subsets “Test 3”.
- The “Test 3” pictures plus the pictures at (45${}^{\circ }$, 0${}^{\circ }$) and (−45${}^{\circ }$, 0${}^{\circ }$) for the SCFace database and the pictures at (135${}^{\circ }$, 0${}^{\circ }$) and (−135${}^{\circ }$, 0${}^{\circ }$) for the FRMDB. We called these subsets “Test 4”. In fact, the “Test 4” includes all the pictures with 0${}^{\circ }$ on the vertical plane of the proposed dataset.
- All the 9 mugshots for the SCFace database, and the “Test 4” pictures plus all the mugshots with a 30${}^{\circ }$ angle on the vertical plane for the FRMDB. We called these subsets “Test 5”.
- All 28 mugshots of the FRMDB. We call this subset “Test 6”.

Concerning the face images from the security cameras to be recognized, from the SCFace database, we took the pictures acquired at 1 m distance from the subject with the 5 color cameras. We excluded three subjects because their face is mostly occluded by their hair (i.e., the tests are based on 635 face images, 5 for each unique subject), using the Multi-Task Cascaded Convolutional Networks (MTCNN) [67] and the Viola–Jones detection framework [41] (implemented in the cascade classifier available in OpenCV) for the face extraction. Instead, for the FRMDB, we used the faces manually cropped from the frames of the security camera videos, as described in Section 3.1. Therefore, there are 5 images for the subject to be recognized in the proposed dataset (consistently with the SCFace database), for a total of 210 face images.

For each subset of mugshots available in the used datasets, we registered the ability of the tested CNNs to identify the subject in the images of the security cameras by logging whether the top-1, top-3, top-5, or top-10 most similar mugshots, and the top-1, top-3, top-5, or top-10 nearest identities contains the correct subject. The CNNs allow computing the face embeddings to describe each face image, whereas the Euclidean distance allows evaluating the similarity between two face embeddings. Using the Euclidean distance as described, we compute the accuracy

- As the number of security camera images for which the correct subject was in the top-1, top-3, top-5, and top-10 most similar mugshots over the total number of security camera images for the most similar mugshots.
- As the number of security camera images for which the correct subject was in the top-1, top-3, top-5, and top-10 nearest identities over the total number of security camera images for the most similar identities.

Obviously, the top-1 identity and the top-1 mugshot overlap.

To give an example of the difference between the nearest mugshots and identities, consider that an image from a security camera contains the face of the “005” identity. In case the nearest mugshots included in Table 3, the correct subject is not in the top-1 ranking because the most similar mugshot is the frontal picture of subject “008”; it is not in the top-3, given that the two following mugshots belong to the “009” identity. Instead, the correct subject belongs to the top-5 ranking, as the first correct mugshot is the fifth. Nevertheless, the correct subject is in the top-3 nearest identities, as “005” is the third recognized identity, after “008” and “009”. Being based on the reference picture, which has the nearest embedding to the face in an image from a security camera, the top-1 is obviously the same regardless considering mugshots or identities.

## 4. Results and Discussion

To validate the FRMDB, provide an initial benchmark for the proposed dataset, and evaluate the impact of using different subsets of mugshots for face recognition in the security camera frames, we ran comparative tests using the methodology described in Section 3. Specifically, we executed the tests on a Jupyter notebook, available in the public GitHub repository of the experiments, in a cloud environment (Google Colab), using Keras 2.8.0 and TensorFlow 2.8.2 to build the CNNs and load the network weights. Hence, in this section, we discuss the results of the SCFace database (Section 4.1) and the proposed dataset (Section 4.2). Furthermore, we list the limitations of the described research (Section 4.3).

### 4.1. Results and Discussion on the SCFace Database

**Figure 5:**
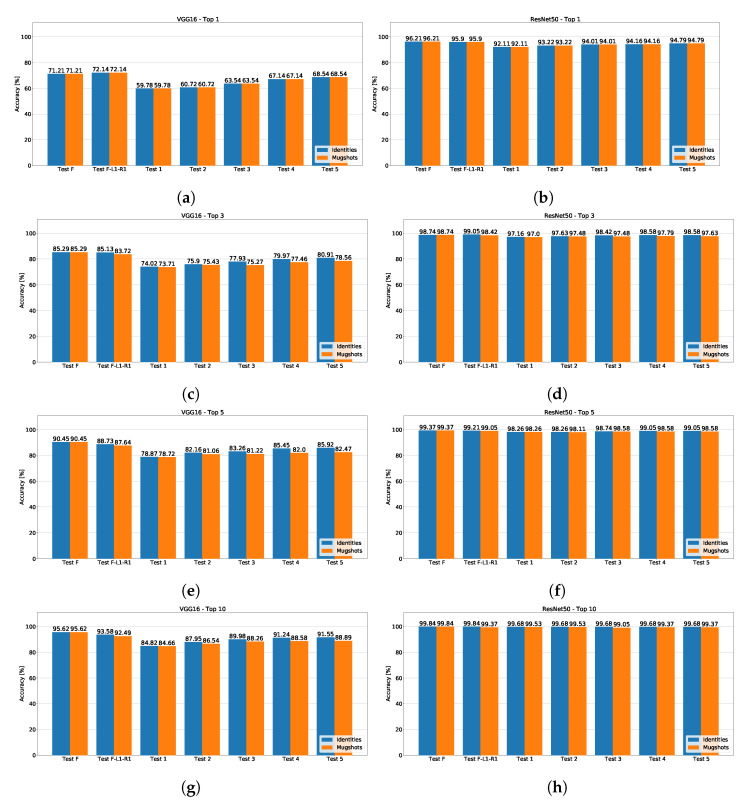
Accuracy measures for ResNet50 (**right**) and VGG16 (**left**) on the SCFace database. The charts include the Top-1 (**a**,**b**), top-3 (**c**,**d**), top-5 (**e**,**f**), and top-10 (**g**,**h**) rankings, in terms of nearest identities (blue) and mugshots (orange).

The performances obtained by ResNet50 and VGG16 are depicted in Figure 5, which includes the recognition accuracy on the images from the five security cameras of the SCFace database, acquired at 1m distance from each unique subject. VGG16 obtained a worse accuracy than ResNet50 in each ranking, regardless of considering the top mugshots or the top identities. Indeed, with ResNet50, the correct subject belongs to the top-10 nearest identities or mugshots in 99% of the pictures from the security cameras, for all the available reference mugshot subsets (Figure 5h). Basically, consideration of the subjects in the top-3 identities makes the face recognition effective (the accuracy is above 98% in all the test but “Test 1” and “2”) when using ResNet50 (Figure 5d). In the top-3 identities, the accuracy obtains a minimum of 97% in the subset composed by the frontal mugshot and the right profile only, i.e., “Test 1”.

Assessing how using mugshots from different POVs affects the face recognition performance, one can notice how the accuracy worsens when the left and right profiles join the reference images (“Test 2”) or even more pictures from different POVs (“Test 3”, “Test 4”, and “Test 5”) become part of the mugshot subsets. In fact, the best results include the frontal mugshot only, as in the “Test F”. The “Top” rankings of VGG16 highlight such phenomenon, as depicted in Figure 5a,c,e,g), irrespective of considering the identities or mugshots. The same trends are available even with ResNet50, which obtains better results, as shown in Figure 5b,d,f,h). Nevertheless, with both CNNs, the frontal mugshot only (“Test F”) achieves the best accuracy with both CNNs. Instead, the test with the frontal picture and the right profile (“Test 1”) achieves the worst accuracy. In other words, the pictures currently available in the database implemented by most police forces obtained the worst results. When adding mugshots from different POVs, the results slightly improve with respect to “Test 1”, without beating those obtained when using the frontal mugshot only (“Test F”). The top-1 accuracy of VGG16 is the only exception to such tendency, as shown in Figure 5a: the subset of mugshots composed by the frontal image (F), the image at 22.5${}^{\circ }$ (R1), and the image at −22.5${}^{\circ }$ (L1), obtained a 72.14% accuracy, against the 71.21% of “Test F”, composed by the frontal mugshot only.

**Figure 6:**
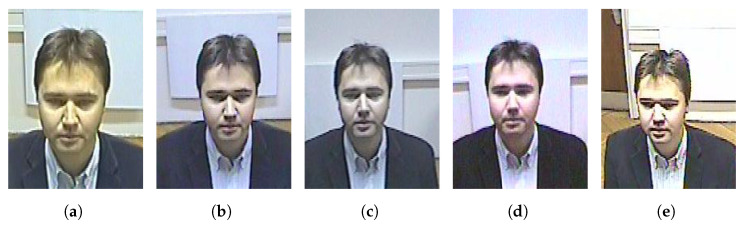
The color pictures from the security cameras acquired at 1 m distance from subject 001 of the SCFace database. The first four images (**a**–**d**) depict a frontal face. The fifth camera (**e**) acquires the face slightly on the right of the depicted person.

Thus, the obtained accuracy indicates that the use of more images acquired from multiple POVs seems not adequate to improve the performance in face recognition. One might conclude that the routine of collecting mugshots during the photo-signalling performed by police forces might not be worth a change because the use of only the frontal mugshot obtained the best results. Nevertheless, we cannot consider such a result conclusive and general. Indeed, such counterintuitive behavior when adding mugshots to the reference subsets might be explained by the security camera pictures in the SCFace database: all the faces from the security cameras are almost frontal, as shown in Figure 6. Instead, the nine posed images, i.e., the mugshots, are acquired from a −90${}^{\circ }$ angle to a 90${}^{\circ }$ angle on the horizontal plane, with a step of 22.5${}^{\circ }$ between two consecutive angles. Therefore, images from multiple POVs added to the frontal face makes the recognition task noisy and the performance worse. Even if the nine posed images in the SCFace database are a perfect archetype of the ideal pictures to evaluate how different mugshot subsets impact a face recognition task, we cannot consider the images from the security cameras fully representative of reality. Indeed, real security cameras frame faces from different casual perspectives.

### 4.2. Results and Discussion on the FRMDB

**Figure 7:**
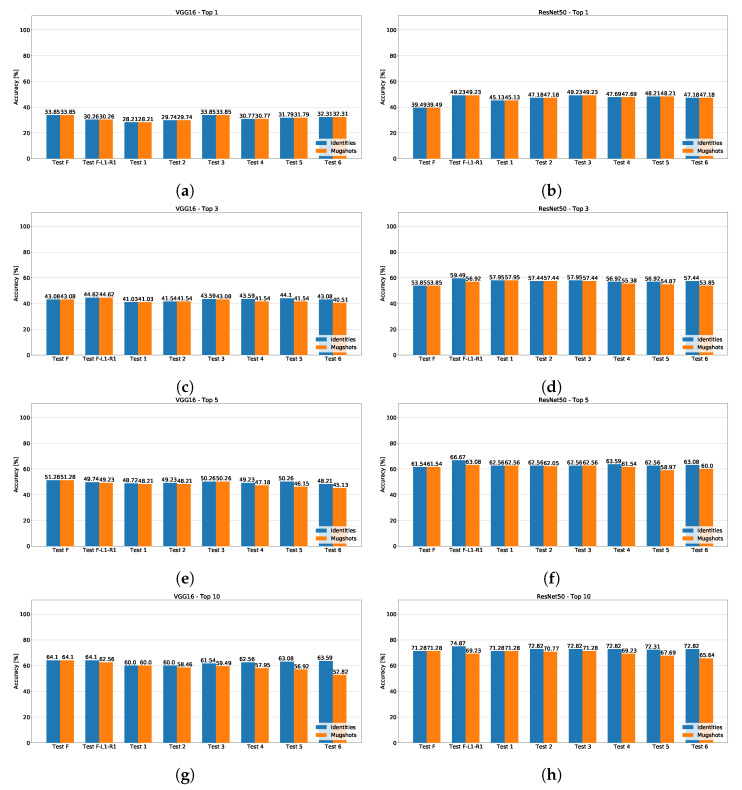
Accuracy measures for ResNet50 (**right**) and VGG16 (**left**) on the proposed database. The charts include the top-1 (**a**,**b**), top-3 (**c**,**d**), top-5 (**e**,**f**), and top-10 (**g**,**h**) rankings in terms of nearest identities (blue) and mugshots (orange).

Figure 7 includes the accuracy results on the proposed dataset. Whereas ResNet50 scores better than VGG16 in all the “Top” rankings even on the proposed dataset, the accuracy is significantly lower than the one obtained on the SCFace for both CNNs. This result suggests that the FRMDB includes challenging features; as shown in Figure 4, the frames of the security cameras are from different perspectives (instead of including frontal faces only as in the SCFace), which is the most challenging feature. In addition, the videos are at low resolution (352 × 288 pixels), emulating low-quality public security cameras, which can include a very small face (such as 85 × 85 pixels) to be recognized from the mugshots. Thus, given the lower accuracy, the proposed dataset seems a better representation of the challenges occurring in real life to recognize faces over security cameras.

Differently from the SCFace database, the subset composed of the frontal picture only (“Test F”) never obtains the best accuracy with ResNet50. Instead, the subset composed of the frontal picture, the picture at (−45${}^{\circ }$, 0${}^{\circ }$) and the picture at (45${}^{\circ }$, 0${}^{\circ }$), i.e., “Test F-L1-R1”, obtains the best results in all the “Top” rankings (Figure 7b,d,f,h). For example, the correct identity is in the top-10 (Figure 7h) nearest identities for the 74.87% of the security camera frames using the pictures in the subset “Test F-L1-R1” as reference images. Such percentage decreases to 71.28% using only the frontal picture as a reference image. Even with VGG16 (Figure 7a,c,e,g), there is not a clear predominance of the subset composed of the frontal image only, differently from the SCFace database. For example, with the subset “Test F-L1-R1”, the correct subject is in the top-3 (Figure 7c) identities and mugshots for 44.62% of the security camera images, whereas with only the frontal image, this percentage decreases to 43.08%.

The tests on the proposed dataset do not exhibit the same trend shown with the SCFace database. Even if the subsets “Test 1”, i.e., the current photo-signaling pictures, and “Test 2” (which adds the left profile to the previous subset) obtains the worst results with both CNNs in both databases, with the proposed dataset, increasing the number of pictures improves the results in some case. Specifically, with VGG16, using all the pictures at 0${}^{\circ }$ and 30${}^{\circ }$ on the vertical plane (“Test 5”) obtains almost the same result as the “Test F-L1-R1”, being capable of recognizing the subject in the top-3 nearest identities (Figure 7c) in 44.1% of the frames.

In general, the results obtained by using subsets of mugshots with more pictures (“Test 3–6”) are better than using the frontal picture or the frontal picture and right profile. Such results and the lower accuracy obtained with respect to the SCFace database validate the proposed dataset as adequate to study the effect of using mugshots from multiple points of view for face recognition in surveillance cameras.

### 4.3. Limitations

The results presented in this paper include some limitations. Concerning the proposed dataset, the main limitation is the number of unique subjects, 39, which might appear low. However, the dataset is not intended to learn face features. For such a task, databases with a proper number of images, up to the million scale, are available in the literature. Instead, the dataset is intended to benchmark face recognition techniques in recognizing subjects in frames of security camera videos using mugshots as reference images. Therefore, the FRMDB can be used for testing, rather than for learning. In addition, the size of the mugshots (972 × 544 pixels) and security camera videos (352 × 288 pixels) might seem small. However, footage from CCTV is usually low-resolution and low-quality to the point that quality enhancement techniques based on DL are emerging [68]. Therefore, we find the proposed dataset representative of real life. In addition, despite the described limitations, the proposed database overcomes the existing literature about face recognition databases by proposing face images systematically taken from different POVs to be compared with images from surveillance cameras. To the best of our knowledge, the FRMDB is the first face recognition database designed with such a purpose.

Concerning the presented results, the tested CNNs are based on the results of the research in face recognition presented in the scientific literature, as explained in Section 2. However, a systematic study of alternative models as well as a comparison of more datasets should be performed to obtain more general insights into the impact that the use of different subsets of mugshots has in face recognition in frames from security cameras. Nevertheless, the tests presented in this paper add to the existing literature an evaluation of the impact of the use of mugshots from multiple POVs in face recognition tasks. To the best of our knowledge, this is the first attempt to fill such a gap in the face recognition research.

## 5. Conclusions

We presented the FRMDB, i.e., a dataset including 28 mugshots pictures and 5 videos from security cameras of 39 unique subjects. The proposed dataset is intended to benchmark face recognition techniques for the identification of the subjects in the videos using the available mugshots.

On the proposed dataset, as well as on the SCFace database, we tested two well-established CNNs, ResNet50 and VGG16, pre-trained on the VGGFace and VGGFace2 datasets for the extraction of facial features. Such experiments allow drawing the following main conclusions:

- The proposed dataset is adequate to benchmark face recognition techniques for the identification of subjects in the videos using mugshots, taking into account different points of view. The lower accuracy with respect to the SCFace database highlighted the challenging nature of the dataset. In addition, the subset of mugshots composed of the frontal face only did not show the same predominance scored on the SCFace, as the FRMDB includes surveillance videos from multiple points of view.
- With both datasets, the traditional photo-signaling pictures, i.e., the frontal image and the right profile, are outperformed by other subsets of mugshots. Specifically, with the proposed FRMDB, the subset composed of the frontal picture and the pictures at ±45${}^{\circ }$ on the horizontal plane achieves the best accuracy in most of the tests.
- Further research is needed to obtain results about an ideal number of mugshots, looking for a compromise with the need for additional tools (and storage space) necessary for law enforcement agencies to collect more mugshots pictures. For more general results, more techniques need to be tested, including those for Pose-Invariant Face Recognition (PIFR) and pose estimation, in order to pick the mugshot with the pose nearest to the security camera frames before the comparison.

Future works on the proposed dataset will address the described limitations by adding more subjects, with videos at higher resolution, in order to have more variability and therefore build a database even more representative of the video surveillance in real life.

## Figures and Tables

**Table 1:** A summary of the features of the face databases discussed in Section 2.1 compared to the proposed dataset. The one proposed in this paper is the only dataset including mugshots from multiple POVs, both on the horizontal and the vertical plane, coupled with videos from security cameras taken from multiple points of view.

Database	# Subjects	# Face Images	Posed/In the Wild	Multiple POVs (°)	Images/Videos From Security Cams	Availability
AT&T [36]	40	400 (grayscale)	Posed	none	none	Not available
LFW [38,39]	5749	13,233 (color)	In the wild	none	none	Open-access
YouTube Faces [40]	1688	3425 (color videos)	In the wild	none	none	Open-access
CASIA-Webface [42]	10,575	494,414 (color)	In the wild	none	none	Upon request
Megaface [43,44]	672,057	4.7 million (color)	In the wild	none	none	Not available
VGGFace [32]	2622	982,803 (color)	In the wild	none	none	Open-access
VGGFace2 [33]	9131	3.31 million (color)	In the wild	none	none	Open-access
FERET [45,46]	1199	14,051 (color)	Posed	Horizontal plane: −60°, −40°, −25°, −15°, 0°, 15°, 25°, 40°, 60° Vertical plane: none	none	Upon request
MPI [47]	200	1400 (color)	Posed	Horizontal plane: from −90° to +90°, 30° step Vertical plane: none	none	Not available
Extended Yale [48,49]	28	16,128 (grayscale)	Posed	Horizontal plane: 0°, 12°, 24° Vertical plane: none	none	Open-access
KFDB [50]	1000	52,000 (color)	Posed	Horizontal plane: from −45° to +45°, 15° step Vertical plane: none	none	Not available
CAS-PEAL [51]	1040	30,900 (color)	Posed	Horizontal plane: from −67.5° to +67.5°, 22.5° step Vertical plane: −30° to +30°, 30° step	none	Upon request
Multi-PIE [52]	337	755,370 (color)	Posed	Horizontal plane: from −90° to +90°, 15° step Vertical plane: 2 pictures on a different unknown angle	none	Upon request
NIST MID [53]	1573	3288 (color)	Posed	Horizontal plane: −90°, 0°, +90° Vertical plane: none	none	Upon request
ChokePoint [54]	25–29	48 (color videos)	Security Cams	Horizontal plane: 3 unknown angles Vertical plane: none	48 Videos from 3 POVs in total	Open-access
SCFace [29]	130	4160 (color and IR)	Posed + Security Cams	Horizontal plane: from −90° to +90°, 22.5° step Vertical plane: none	23 Frontal Face Images per subject	Upon request
**FRMDB (proposed)**	**39**	**1092 (color)** **195 (color videos)**	**Posed +** **Security Cams**	**Horizontal plane: from −135° to +135°, 45° step** **Vertical plane: −60° to +30°, 30° step**	**5 Videos from multiple** **POVs per subject**	**Open-access**

**Table 2:** The subsets of mugshots from the SCFace database and the FRMDB used as reference images in the tests. The table lists the name we give to each subset and, for each database, the angles from which the included mugshots were taken as a couple (h,v), where *h* is the angle on the horizontal plane and *v* is the angle on the vertical plane.

Subset Name	Mugshots (SCFace)	Mugshots (FRMDB)
“Test F”	(0${}^{\circ }$, 0${}^{\circ }$)	(0${}^{\circ }$, 0${}^{\circ }$)
“Test F-L1-R1”	(0${}^{\circ }$, 0${}^{\circ }$), (−22.5${}^{\circ }$, 0${}^{\circ }$), (22.5${}^{\circ }$, 0${}^{\circ }$)	(0${}^{\circ }$, 0${}^{\circ }$), (−45${}^{\circ }$, 0${}^{\circ }$), (45${}^{\circ }$, 0${}^{\circ }$)
“Test 1”	(0${}^{\circ }$, 0${}^{\circ }$), (90${}^{\circ }$, 0${}^{\circ }$)	(0${}^{\circ }$, 0${}^{\circ }$), (90${}^{\circ }$, 0${}^{\circ }$)
“Test 2”	(0${}^{\circ }$, 0${}^{\circ }$), (90${}^{\circ }$, 0${}^{\circ }$), (−90${}^{\circ }$, 0${}^{\circ }$)	(0${}^{\circ }$, 0${}^{\circ }$), (90${}^{\circ }$, 0${}^{\circ }$), (−90${}^{\circ }$, 0${}^{\circ }$)
“Test 3”	(0${}^{\circ }$, 0${}^{\circ }$), (90${}^{\circ }$, 0${}^{\circ }$), (−90${}^{\circ }$, 0${}^{\circ }$), (77.5${}^{\circ }$, 0${}^{\circ }$), (−77.5${}^{\circ }$, 0${}^{\circ }$)	(0${}^{\circ }$, 0${}^{\circ }$), (90${}^{\circ }$, 0${}^{\circ }$), (−90${}^{\circ }$, 0${}^{\circ }$), (45${}^{\circ }$, 0${}^{\circ }$), (45${}^{\circ }$, 0${}^{\circ }$)
“Test 4”	(0${}^{\circ }$, 0${}^{\circ }$), (90${}^{\circ }$, 0${}^{\circ }$), (−90${}^{\circ }$, 0${}^{\circ }$), (77.5${}^{\circ }$, 0${}^{\circ }$), (−77.5${}^{\circ }$, 0${}^{\circ }$), (45${}^{\circ }$, 0${}^{\circ }$), (−45${}^{\circ }$, 0${}^{\circ }$)	(0${}^{\circ }$, 0${}^{\circ }$), (135${}^{\circ }$, 0${}^{\circ }$), (−135${}^{\circ }$, 0${}^{\circ }$), (90${}^{\circ }$, 0${}^{\circ }$), (−90${}^{\circ }$, 0${}^{\circ }$), (45${}^{\circ }$, 0${}^{\circ }$), (45${}^{\circ }$, 0${}^{\circ }$)
“Test 5”	(0${}^{\circ }$, 0${}^{\circ }$), (90${}^{\circ }$, 0${}^{\circ }$), (−90${}^{\circ }$, 0${}^{\circ }$), (77.5${}^{\circ }$, 0${}^{\circ }$), (−77.5${}^{\circ }$, 0${}^{\circ }$), (45${}^{\circ }$, 0${}^{\circ }$), (−45${}^{\circ }$, 0${}^{\circ }$), (−22.5${}^{\circ }$, 0${}^{\circ }$), (22.5${}^{\circ }$, 0${}^{\circ }$)	(0${}^{\circ }$, 0${}^{\circ }$), (135${}^{\circ }$, 0${}^{\circ }$), (−135${}^{\circ }$, 0${}^{\circ }$), (90${}^{\circ }$, 0${}^{\circ }$), (−90${}^{\circ }$, 0${}^{\circ }$), (45${}^{\circ }$, 0${}^{\circ }$), (45${}^{\circ }$, 0${}^{\circ }$), (0${}^{\circ }$, 30${}^{\circ }$), (135${}^{\circ }$, 30${}^{\circ }$), (−135${}^{\circ }$, 30${}^{\circ }$), (90${}^{\circ }$, 30${}^{\circ }$), (−90${}^{\circ }$, 30${}^{\circ }$), (45${}^{\circ }$, 30${}^{\circ }$), (45${}^{\circ }$, 30${}^{\circ }$)
“Test 6”	None	(0${}^{\circ }$, 0${}^{\circ }$), (135${}^{\circ }$, 0${}^{\circ }$), (−135${}^{\circ }$, 0${}^{\circ }$), (90${}^{\circ }$, 0${}^{\circ }$), (−90${}^{\circ }$, 0${}^{\circ }$), (45${}^{\circ }$, 0${}^{\circ }$), (45${}^{\circ }$, 0${}^{\circ }$), (0${}^{\circ }$, 30${}^{\circ }$), (135${}^{\circ }$, 30${}^{\circ }$), (−135${}^{\circ }$, 30${}^{\circ }$), (90${}^{\circ }$, 30${}^{\circ }$), (−90${}^{\circ }$, 30${}^{\circ }$), (45${}^{\circ }$, 30${}^{\circ }$), (45${}^{\circ }$, 30${}^{\circ }$), (0${}^{\circ }$, 60${}^{\circ }$), (135${}^{\circ }$, 60${}^{\circ }$), (−135${}^{\circ }$, 60${}^{\circ }$), (90${}^{\circ }$, 60${}^{\circ }$), (−90${}^{\circ }$, 60${}^{\circ }$), (45${}^{\circ }$, 60${}^{\circ }$), (45${}^{\circ }$, 60${}^{\circ }$), (0${}^{\circ }$, −30${}^{\circ }$), (135${}^{\circ }$, −30${}^{\circ }$), (−135${}^{\circ }$, −30${}^{\circ }$), (90${}^{\circ }$, −30${}^{\circ }$), (−90${}^{\circ }$, −30${}^{\circ }$), (45${}^{\circ }$, −30${}^{\circ }$), (45${}^{\circ }$, −30${}^{\circ }$)

**Table 3:** An example of how the accuracy is computed in this paper: with the following nearest results in terms of Euclidean distance, if the subject to be recognized is “005”, the correct result is in the top-5 most similar mugshots and in the top-3 nearest identities (after “008” and “009”, the third recognized identity is “005”).

**1.** 008 (0°, 0°)	**6.** 001 (0°, 0°)
**2.** 009 (0°, 0°)	**7.** 005 (45°, 0°)
**3.** 009 (45°, 0°)	**8.** 005 (45°, 30°)
**4.** 008 (−45°, 30°)	**9.** 002 (0°, 0°)
**5.** 005 (0°, 0°)	…

## Data Availability

The dataset proposed and used in this study is publicly available at https://github.com/airtlab/face-recognition-from-mugshots-database, accessed on the 30 December 2022. The source code of the experiments performed on the proposed dataset is publicly available at https://github.com/airtlab/tests-on-the-FRMDB, accessed on the 30 December 2022.
